# The Dietetic Gazette

**Published:** 1890-06

**Authors:** 


### The Dietetic Gazette, P. O. Box 2898, New York.

The editorial article of the May issue has been prepared by J. Lewis Smith, M. D., Clinical Professor of Diseases of Children, in Bellevue Hospital Medical College. With the June number will begin an extended article by J. Lewis Smith, M. D., on the Care and Feeding of Infants, with remarks on The Great Mortality of Infants in the Summer Months, and mode of preventing it.

